# Clinical massage therapy for patients with exercise-induced fatigue: A protocol for systematic review and meta analysis

**DOI:** 10.1097/MD.0000000000020870

**Published:** 2020-06-26

**Authors:** Ke-Lin Zhou, Shuo Dong, Kang Wang, Guo-Bing Fu, Shu-Sheng Cui, Sheng Guo

**Affiliations:** a Dongfang Hospital; b School of Traditional Chinese Medicine, Beijing University of Chinese Medicine; c Beijing Gulou Hospital of Traditional Chinese Medicine, Beijing, China.

**Keywords:** complementary and alternative medicine, exercise-induced fatigue, massage, protocol

## Abstract

**Background:**

Exercise-induced fatigue (EF) has been a major area of interest within the field of sports and clinical medicine. Implemented on people's skin, muscles, and joints as an important part of complementary and alternative medicine , massage therapy has a positive effect on the recovery of EF and sports injuries. In this systematic review, we aim to evaluate the effectiveness and safety of massage therapy for patients with EF.

**Methods:**

We will search the following electronic databases for randomized controlled trials to evaluate the effectiveness and safety of massage therapy in treating EF: China National Knowledge Infrastructure, Wanfang and PubMed Database, Cochrane Central Register of Controlled Trials, Cumulative Index of Nursing and Allied Health Literature, Excerpta Medica database, and Medical Literature Analysis and Retrieval System Online. Each database will be searched from inception to May 2020. The entire process will include study selection, data extraction, risk of bias assessment and meta-analyses.

**Results:**

This proposed study will evaluate the effectiveness and safety of massage therapy for patients with EF. The outcomes will include change in fatigue relief and adverse effect.

**Conclusions:**

This proposed systematic review will evaluate the existing evidence on the effectiveness and safety of massage therapy for patients with EF.

**Dissemination and ethics:**

The results of this review will be disseminated through peer-reviewed publication. Because all of the data used in this systematic review and meta-analysis has been published, this review does not require ethical approval. Furthermore, all data will be analyzed anonymously during the review process.

## 1 Introduction

Exercise-induced fatigue (EF) has been a major area of interest within the field of sports and clinical medicine.^[1]^ People with EF cannot sustain their function at a certain level and the organs cannot maintain the predetermined exercise intensity.^[2,3]^ The characteristics of competitive sports challenging human physiological limits lead to unavoidable exercise fatigue. If EF is unable to be eliminated or relieved appropriately, excessive fatigue is bound to affect the athletes’ training scores and cause sports injuries.^[4,5]^ People with fatigue usually suffer from insomnia and dreams, memory loss, lack of energy, and inattention, resulting in decreased work efficiency and exercise capacity.^[6,7]^ It is reported that fatigue is not only related to cardiovascular diseases, mental diseases and cancer, etc, but also induces diseases such as hypertension, diabetes, and cerebral hemorrhage.^[8,9]^ What is more, fatigue is also the main causes of many accidents.^[10]^

Complementary and alternative medicine(CAM) is considered as an adjunct to treat chronic or serious diseases and to self-manage long-term health complaints.^[11]^ Reviewing the global literature, CAM is frequently used in the treatment of back or neck pain, severe headaches or migraines, depression, insomnia, stomach or digestive system-related problems and allergies.^[12,13]^ Massage therapy is one of the widely employed CAM interventions in the world. As a sport therapy implemented on human's skin, muscles and joints, Tuina (massage) has unique advantages in the field of medicine. In recent years, medical industries related to “Massage” in the United States has grown rapidly, increasing by 19% between 2008 and 2009. After 2009, American consumers spend 4 to 6 billion dollars on “Massage” each year.^[14]^ Recent studies have found that Tuina has a positive effect on the recovery of exercise fatigue and injury, as well as the mental health.^[15]^ Massage can activate muscle fibers, relieve muscle tension, reduce swelling, pull soft tissue, improve flexibility, loosen adhesions and accelerate blood circulation and lymph flow and speed up healing of injuries. What is more, massage can alleviate athletes’ physical pain and psychological tension or anxiety.^[4,16]^

This review aims to systematically review all randomized controlled trials (RCTs) to assess the effectiveness and safety of massage treatment for patients with EF.

## 2 Materials and methods

This systematic review protocol has been registered on PROSPERO (ID: CRD42020153308). The protocol follows the Cochrane Handbook for Systematic Reviews of Interventions and the Preferred Reporting Items for Systematic Reviews and Meta-Analysis Protocol statement guidelines.^[17]^ We will describe the changes in our full review if needed.

## 3 Inclusion criteria for study selection

### 3.1 Type of studies

This review will include clinical RCTs of clinical massage therapy for EF patients without any language or publication status restrictions. Non-RCTs, quasi-RCTs, case series, case reports, crossover studies, uncontrolled trials, and laboratory studies will not be included.

### 3.2 Type of participants

Participants, male or female, of any age with a diagnosis of EF will be included. The diagnosis is based on International Statistical Classification of Diseases and Related Health Problems, 10th revision (ICD-10) proposed criteria and required evidence from the history, physical examination, and laboratory findings that the fatigue was a result of exercise or sports.

### 3.3 Type of interventions

Interventions will include any type of clinically performed massage for improvement of EF. This will include Chinese Massage, Japanese Massage, Thai Massage, Swedish Massage, Tuina, Shiatsu, Remedial Massage, General Massage, Acupressure, Reflexology, Manual Lymphatic Drainage. Studies of EF combined with other interventions such as acupuncture, herbal medicines, qigong, and yoga will be considered for exclusion.

Control: Rest, no intervention, standard care, and other body-based practices including exercise techniques, qigong, yoga, and taichi.

### 3.4 Type of outcome measures

#### 3.4.1 Main outcome(s)

Change in fatigue score from baseline to the last available follow-up, using any validated measurement for EF.

Timing and effect measures:

1. Lactic acid
2. Blood urea nitrogen
3. Superoxide dismutase
4. Multidimensional Fatigue Inventory
5. Patient Reported Outcomes Measurement Information System Fatigue-Short Form.
6. Multidimensional Fatigue Symptom Inventory-Short Form
7. Fatigue Symptom Inventory
8. Visual Analogue Scale

#### 3.4.2 Additional outcome(s)

Type, frequency, and severity of adverse effects.

## 4 Search methods for the identification of studies

### 4.1 Electronic searches

We will search the following electronic bibliographic databases for relevant trials:

- China National Knowledge Infrastructure Database (from 1979 to present)
- Wanfang Database (from 1990 to present)
- PubMed Database (from 2000 to present)
- Cochrane Central Register of Controlled Trials
- Cumulative Index of Nursing and Allied Health Literature (from 1937 to present)
- Excerpta Medica database (from 1947 to present)
- Ovid Medical Literature Analysis and Retrieval System Online (from 1946 to present)

There will be no language restrictions.

### 4.2 Data collection and analysis

#### 4.2.1 Study identification

We will use EndNote X9 software (Alfasoft Limited, A.W. house, United Kingdom) to manage the records of searched electronic databases. The initial selection will involve scanning of the titles and abstracts of the retrieved studies. The full text of relevant studies will then be reviewed for study inclusion, in accordance with the inclusion criteria, by 2 authors (K-LZ and SD). Potentially relevant articles will be reviewed independently by 2 authors (K-LZ and KW) to determine if they meet the prespecified criteria. Any disagreement between authors will be resolved by consensus with a third author. The study selection procedure will follow and be recorded in the Preferred Reporting Items for Systematic Reviews and Meta-analysis Protocol flowchart. All the evidence will be assessed by The Grading of Recommendations Assessment, Development and Evaluation.

#### 4.2.2 Data extraction and management

According to the inclusion criteria, a standard data collection form will be made before data extraction. The following data will be extracted by 2 authors (K-LZ and SD):

- General information: research identification, publication year, the title of the study, first author
- Study methods: study design, sample size, randomization method, allocation concealment, blinding, incomplete report or selecting report, other sources of bias
- Participants: inclusion and exclusion criteria
- Intervention: motion details, treatment duration, and frequency
- Control: type of control methods, motion details, treatment duration, and frequency
- Outcomes: included outcome measures

#### 4.2.3 Risk of bias assessment

The risk of bias in included studies will be assessed independently by 2 reviewers (K-LZ and KW) using the Cochrane Risk of Bias Tool, with any disagreements resolved by consensus or by discussion with a third reviewer. All judgments will be fully described, and the conclusions will be presented in the Risk of Bias figures and will be incorporated into the interpretation of review findings, by means of sensitivity analysis. The risk of bias of each domain will be graded as adequate, unclear, or inadequate. We intend to use the concealment of allocation grading in investigation of any heterogeneity and in sensitivity analysis. Other aspects of study quality including the extent of blinding (if appropriate), losses to follow up, non-compliance, whether the outcome assessment was standardized, and whether an intention to treat analysis was undertaken, will be presented in the risk of bias table describing the included studies and will provide a context for discussing the reliability of the results.

#### 4.2.4 Data analysis

We will use Stata Software (StataCorp: College Station, TX, USA) [Computer program] (Version 15.1) to process the meta-analysis. Weighted mean difference will be used for continuous variable data, and the combined statistical effects of these two are combined. The X^2^ test will be adopted to analyze whether there is heterogeneity in each of the included research questions. I^2^ > 50% is a criterion for significant judgment. The fixed effect model is adopted if I^2^ ≤ 50%, which is considered to have homogeneity between the studies. The random effect model is adopted if I^2^ > 50%, which is considered to have heterogeneity among the studies. The effect size is expressed as 95% confidence interval and *P* <.05 is considered to be statistically significant.

**Sensitivity analyses:** heterogeneity may be due to the presence of 1 or more outlier studies with results that conflict with the rest of the studies. We will perform sensitivity analyses excluding outlier studies. In addition, we plan to perform sensitivity analysis to explore the influence of trial quality on effect estimates. The quality components of methodology include adequacy of generation of allocation sequence, concealment of allocation, and the use of intention-to-treat analysis.

**Meta-regression analyses:** if data permits, we will perform the meta-regression analyses.

#### 4.2.5 Publication bias

If sufficient number of trials (more than 10 trials) are found, we will generate funnel plots (effect size against standard error) to investigate publication bias.

#### 4.2.6 Ethics and dissemination

The data used in this systematic review will be collected from published studies. Based on this, the study does not require ethical approval.

## Acknowledgment

The authors would like to thank all the researchers in our working group.

## Author Contributions

KLZ, SD contributed on methodology and are the guarantors of the review.

KLZ, SD, KW and SG contributed on data search, analysis, and statistics.

**Data curation:** Ke-Lin Zhou, Shuo Dong, Kang Wang.

**Funding acquisition:** Sheng Guo.

**Methodology:** Ke-Lin Zhou, Shuo Dong.

**Project administration:** Shuo Dong.

**Resources:** Shu-Sheng Cui, Sheng Guo.

**Software:** Ke-Lin Zhou.

**Supervision:** Guo-Bing Fu.

**Writing – original draft:** Ke-Lin Zhou, Shuo Dong.

**Writing – review & editing:** Kang Wang, Guo-Bing Fu, Shu-Sheng Cui, Sheng Guo.
